# Competence By Design: a transformational national model of time-variable competency-based postgraduate medical education

**DOI:** 10.5334/pme.1096

**Published:** 2024-03-18

**Authors:** Jason R. Frank, Jolanta Karpinski, Jonathan Sherbino, Linda S. Snell, Adelle Atkinson, Anna Oswald, Andrew K. Hall, Lara Cooke, Susan Dojeiji, Denyse Richardson, Warren J. Cheung, Rodrigo B. Cavalcanti, Timothy R. Dalseg, Brent Thoma, Leslie Flynn, Wade Gofton, Nancy Dudek, Farhan Bhanji, Brian M.-F. Wong, Saleem Razack, Robert Anderson, Daniel Dubois, Andrée Boucher, Marcio M. Gomes, Sarah Taber, Lisa J. Gorman, Jane Fulford, Viren Naik, Kenneth A. Harris, Rhonda St. Croix, Elaine van Melle

**Affiliations:** 1 Centre for Innovation in Medical Education and Professor, Department of Emergency Medicine, Faculty of Medicine, University of Ottawa, ON, Canada; 2 Department of Medicine, University of Ottawa, Ottawa, ON, Canada; 3 Competency Based Medical Education, University of Ottawa, Ottawa, ON, Canada; 4 Royal College of Physicians and Surgeons of Canada, Ottawa, ON, Canada; 5 McMaster University, Hamilton, ON, Canada; 6 Medicine and Health Sciences Education, McGill University, Montreal, QC, Canada; 7 Department of Paediatrics, Temerty Faculty of Medicine, University of Toronto, Toronto, ON, Canada; 8 Department of Medicine, Faculty of Medicine and Dentistry, University of Alberta, Edmonton, AB, Canada; 9 Competency Based Medical Education, University of Alberta, Edmonton, AB, Canada; 10 Department of Emergency Medicine, University of Ottawa, Ottawa, ON, Canada; 11 Division of Neurology, Department of Clinical Neurosciences, Cumming School of Medicine, University of Calgary, Calgary, AB, Canada; 12 Physical Medicine and Rehabilitation, University of Ottawa, Ottawa, ON, Canada; 13 Department of Physical Medicine and Rehabilitation, Queen’s University, Kingston, ON, Canada; 14 Department of Medicine, University of Toronto, Toronto, ON, Canada; 15 HoPingKong Centre, University Health Network, Toronto, ON, Canada; 16 Division of Emergency Medicine, University of Toronto, Toronto, ON, Canada; 17 Emergency Medicine, University of Saskatchewan, Saskatoon, SK, Canada; 18 Departments of Psychiatry and Family Medicine, and Co-Director Master of Health Sciences Education, Queen’s University, Kingston, ON, Canada; 19 Department of Surgery (Division of Orthopedic Surgery), The Ottawa Hospital and University of Ottawa, Ottawa, ON, Canada; 20 Department of Medicine (Division of Physical Medicine & Rehabilitation) and The Ottawa Hospital, University of Ottawa, Ottawa, Ontario, Canada; 21 Faculty of Medicine and Health Sciences, McGill University, Montreal, QC, Canada; 22 Centre for Quality Improvement and Patient Safety, University of Toronto, Toronto, Canada; 23 Centre for Health Education Scholarship, University of British Columbia and BC Children’s Hospital, Vancouver, BC, Canada; 24 Northern Ontario School of Medicine University, Sudbury, ON, Canada; 25 Department of Anesthesiology and Pain Medicine, University of Ottawa, Ottawa, ON, Canada; 26 Department of Medicine (Division of Endocrinology), Universitéde Montréal, Montréal, QC, Canada; 27 Department of Pathology and Laboratory Medicine, University of Ottawa, Ottawa, ON, Canada; 28 Office of Standards and Assessment, Royal College of Physicians and Surgeons of Canada, Ottawa, ON, Canada; 29 Canadian Internet Registration Authority, Canada; 30 Medical Council of Canada, Ottawa, ON, Canada; 31 Royal College of Physicians and Surgeons of Canada, Canada; 32 Emeritus, Western University, Canada; 33 Learning and Connecting at the Royal College of Physicians and Surgeons of Canada, Canada; 34 Department of Family Medicine, Queen’s University, Kingston, ON, Canada

## Abstract

Postgraduate medical education is an essential societal enterprise that prepares highly skilled physicians for the health workforce. In recent years, PGME systems have been criticized worldwide for problems with variable graduate abilities, concerns about patient safety, and issues with teaching and assessment methods. In response, competency based medical education approaches, with an emphasis on graduate outcomes, have been proposed as the direction for 21st century health profession education. However, there are few published models of large-scale implementation of these approaches. We describe the rationale and design for a national, time-variable competency-based multi-specialty system for postgraduate medical education called Competence by Design. Fourteen innovations were bundled to create this new system, using the Van Melle Core Components of competency based medical education as the basis for the transformation. The successful execution of this transformational training system shows competency based medical education can be implemented at scale. The lessons learned in the early implementation of Competence by Design can inform competency based medical education innovation efforts across professions worldwide.

## Introduction

Postgraduate medical education (PGME) has been described as an essential societal enterprise that prepares physicians to achieve the level of competence needed to practise and serve society [1]. Without an effective PGME system, a population may lack a sufficient health workforce, or have a cadre of physicians who are not adequately prepared for practice. The 20^th^ century model of medical education, heavily influenced by Osler, Halsted, and Flexner, evolved out of an apprenticeship model that progressively incorporated more educational structure over decades [2, 3]. However, this model has been criticized as inadequate for the 21 st century [4,5,6,7,8,9,10] and in need of greater attention to social accountability [11, 12]. In response, new outcomes-oriented and competency-based approaches have been endorsed [13,14,15,16,17,18]. Worldwide, competency based medical education (CBME) has become a major transformational movement in the health professions [19,20,21,22,23].

CBME has been defined as “an outcomes-based approach to the design, implementation, assessment and evaluation of an education program using an organizing framework of competencies” [18]. This approach to health professions education (HPE) extends back to a major report by the World Health Organization [24], later further developed by many authors and organizations. The International CBME Collaborators have proposed five elements (the van Melle Core Components) of a modern CBME model:

1. Training outcomes organized as a competency framework for graduates
2. Defined progression of training from novice to expert
3. Tailored learning experiences to meet the needs of learners
4. Teaching focused on competency achievement
5. Programmatic assessment [25]

PGME systems in many countries have moved to adopt CBME [26,27,28,29,30,31]. Driving this movement are a number of concerns about contemporary training and opportunities to enhance PGME design. Patient safety risks [32,33,34], variability in graduate competence [35,36,37,38], issues with transitions to, within, and from PGME [39,40,41,42,43], inadequate supervision and insufficient direct observation of trainee work [44,45,46,47,48,49,50,51,52], concerns with workplace-based assessments and promotion decisions [53,54,55,56,57,58,59], lack of equity in clinical assessments [60, 61], and little or poor feedback [62,63,64,65,66,67] are all examples of important recurring challenges with PGME that education leaders have sought to address. At the same time, innovations and developments such as programmatic assessment [68,69] entrustable professional activities (EPAs) [70, 71], new coaching feedback models [72, 73], deliberate practice and mastery learning [74, 75], Competence Committees [76, 77], assessment software [78,79,80], learning analytics [81], and novel approaches to accreditation [82] all present significant opportunities for better PGME through the implementation of the best evidence in medical education (See Table 1).

**Table 1:** Drivers of the Competence by Design project.


ISSUES OF CONCERN IN PGME SYSTEM	OPPORTUNITIES FOR PGME SYSTEM ENHANCEMENT

Public expectations for greater social accountability of health professions and their education systems [11, 12] Calls for greater focus on outcomes of training [14,15,16,17,18] Patient safety concerns with care provided during and after postgraduate training [32,33,34] Evidence of unacceptable variability in the competence of medical graduates [35,36,37,38]	Recommendations to shift to outcomes-oriented, competency-based systems have been made by major medical organizations (e.g., World Health Organization) [90,91,92] There are successful CBME implementations to build upon (e.g., Toronto Orthopedics [93], CFPC [94], ACGME [91])

Little direct observation of trainees at work Incidents of inadequate supervision of trainees [44,45,46,47,48,49,50,51,52]	Use of entrustable professional activities allow more faculty to provide better input on trainee progress [70, 71]

Failure to address identified weaknesses in trainees (“failure to fail”) Certification examination failures Promotion despite evidence of gaps or unreadiness for practice Concerns about promotion decision-making Concerns about inadequate workplace-based assessments Few supervisors involved in workplace assessment [53,54,55,56,57,58,59]	Use of programmatic assessment can enhance assessment decisions [68, 69, 95] Application of learning analytics to medical education allows for new insights into trainee progression toward competence [81, 96]

Reports of workplace assessments perceived as burdensome [53]	Development of electronic portfolio software allows digitization and documentation of assessments [78,79,80]

Reports of inadequate quality and frequency of the feedback given to trainees [62,63,64,65,66,67]	New coaching models have been developed for medical education [72, 73] A CanMEDS framework update incorporates developmental milestones that can be used as scaffolding for supervisor feedback [88]

Reports of trainee anxiety with workplace assessment [97, 98]	Growth mindset may enhance learning [99,100,101]

Issues of transitions to postgraduate training, transition to senior trainee responsibilities, and transitions to practice [39,40,41,42,43, 103]	Mastery learning methods enhance learning [74, 75] Stages of training may allow for explicitly addressing transitions into and out of PGME [25, 103]

Reports of trainee disengagement [104]	Greater trainee engagement with training enhances learning outcomes [105]

Reports of assessment inequity [60, 61] Concerns that *assessment of learning* approaches overemphasize seeking trainees with problems instead of trainee development [77]	A developmental view of training allows for tailoring training and assessment to ensure every trainee progresses to competence (*assessment for learning*) [68]

Program reviews inordinately focused on process measures that may not enhance training [106]	New accreditation systems place greater emphasis on program outcomes and continuous improvement [106, 107] Application of learning analytics to medical education allows for new insights into program performance [108]


*ACGME* Accreditation Council for Graduate Medical Education; *CBME* competency based medical education; *CFPC* College of Family Physicians of Canada; *PGME* postgraduate medical education.

Using the Core Components, CBME designs can address these issues and opportunities. CBME shifts the emphasis from time spent in training to competencies achieved by graduates. A clear statement of the levels and types of competencies required of a graduate directs the attention of learners and teachers to a shared mental model of competence [83]. A developmental approach to attainment of competence is reflected in deliberately sequenced training experiences and coaching feedback. More frequent and better quality feedback enhances learning and trainee satisfaction. Programmatic assessment, with many data points contributed by a variety of assessors and tools, allows for better informed and more equitable decision-making about learner progress. Combined, these CBME design elements have the potential to ensure trainees are truly prepared for each stage of training, providing safe and effective care [25].

These changes to longstanding HPE designs have led to criticisms that CBME is a set of assertions with no evidence base, that the underlying assumptions are invalid, and that there is a lack of proof of concept of CBME at a national scale [84, 85]. While there are large-scale CBME implementation projects underway around the world, few have been described in the literature. Without an evidence base describing CBME implementation in a variety of settings, these outcome-focused approaches may be regarded as aspirational, theoretical, or unfounded.

We describe the transformational change of a national PGME environment to a multi-specialty, time-variable competency-based system. The van Melle Core Components of CBME were used as the basis of specialty PGME. This major system reform project was called Competence by Design (CBD) [86] to distinguish it from the previous system, which was based on achieving competence by time-based training. This paper provides an overview of the rationale, drivers, and the bundle of educational interventions that formed the CBD national innovation. Accompanying papers in this special collection explore specific aspects of Competence by Design, while this one focuses on the aims and innovations involved in putting CBME theory into practice.

## Context

In Canada, the PGME system is an interwoven network of university medical schools, academic hospitals, community clinical teaching centres, government funders, and regulatory bodies [87]. The Royal College of Physicians and Surgeons of Canada (Royal College) is a specialty standards body created by an act of federal Parliament to oversee specialty medicine standards, accreditation, certification, and maintenance of competence outside of family medicine. In the contemporary landscape, the Royal College partners with all stakeholders and institutions in the PGME system to carry out its functions. In this paper, we present the early stages of a transformational medical education change from the perspective of the design, policy, and standards teams of the Royal College who were involved at the time. Thousands of other individuals contributed to and would also have perspectives on this transformation.

Twentieth-century Canadian PGME had a typical North American design. Following medical school graduation, trainees entered a system of time-based training in clinical settings. Canadian training is overseen by three collaborating medical Colleges: the Royal College (67 specialties and subspecialties), the College of Family Physicians of Canada (family medicine), and the Collège des médecins du Québec (all disciplines recognized in the region of Quebec). Directly from medical school, trainees outside of family medicine entered into Royal College programs leading to certification in primary specialties. All Canadian specialties and subspecialties were structured around the CanMEDS competency framework [88, 89]. Training consisted of immersion in specific clinical services typically from four to 52 weeks, as well as structured regular instruction in classrooms, skills workshops, simulation sessions, or laboratories. Experiences were selected to provide opportunities for trainees to acquire the defined competencies relevant to the specialty of training, prepare for certification examinations, provide needed clinical services, and meet all the criteria for credentials. Assessment most commonly entailed a CanMEDS-based retrospective form completed by a single supervisor at the end of every four-week block of training. Some training sites incorporated other assessment methods (e.g., objective structured clinical examinations [OSCEs]) on an ad hoc basis. Typically, a Royal College trainee would rotate through 13 blocks each year for four to six years before writing a final, high-stakes Royal College specialty examination. Successful trainees would then be certified in their specialty, enabling them to move to practice, begin a subspecialty training program, or undertake less-structured further fellowship training.

## Drivers for system change

At the time of its development, CBD was driven by the Royal College’s commitment to continuous improvement in the Canadian PGME system, as a fiduciary duty to those served by the medical profession. The Royal College, along with other stakeholders, scanned the environment for areas of concern and opportunities to enhance the training of future physicians. These are summarized in Table 1.

## Development process

**Figure 1:**
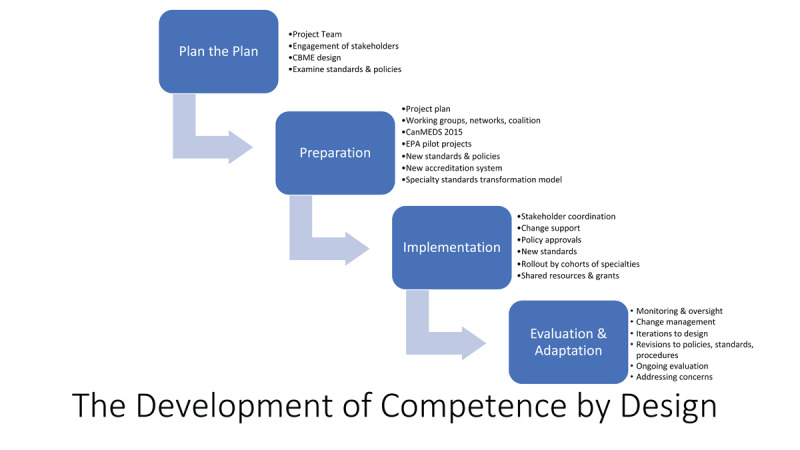
Phases of Competence by Design development.

Canadian PGME had a history of major reforms, including the incorporation of the CanMEDS competency framework going back to 1990 [109,110,111]. Not all of these proposed reforms were successful [112], so the desire to improve the PGME system through CBME was organized into a formal system-wide project to support its success. The Royal College launched a major institutional project group to develop CBD, including teams responsible for education strategy, specialty standards, CanMEDS, faculty development, accreditation, policy, assessment, finance, IT, communications, and governance. The CBD project was organized into four phases (see Figure 1 and Supplement A). To do its work, the project group adopted six principles applied to the PGME system:

### Organizational alignment and support

To execute this project effectively, the involved organizations themselves would need to be transformed to align with the initiative. Therefore, the Royal College and its medical school and organizational partners created formal project teams, working groups, shared governance bodies, change strategies, and new policies that facilitated the creation of the new competency-based PGME system [113].

### Stakeholder engagement and co-production

As this was recognized as a transformational change in a long-established system, the project group prioritized early and extensive stakeholder engagement [114, 115]. Deans, government representatives, medical students, postgraduate trainees, senior education leaders, front-line teachers, education administrators, medical regulators, and many others were invited to co-produce CBD as a community. Engagement and support varied, but the majority of stakeholders supported the change effort.

### Iterative community development and rollout

Early on, it was decided that this large transformational change required an iterative approach. Educational and policy designs were brainstormed in a series of summits beginning in 2010, which were then widely circulated among stakeholders for comment and improvement before implementation. Similarly, specialties and subspecialties (e.g., Anesthesiology, Medical Oncology, Otolaryngology-Head and Neck Surgery) were invited to be early adopters and volunteer to join the first cohorts of disciplines to implement the new model. Lessons learned from each step of the journey informed changes for the next cohort and the whole system [116].

### Resource sharing

As this was a large, systemic change, it was recognized early that additional education resources would be needed. New faculty development resources (conferences, webinars, videos, templates) were developed [86]. Grants were established to support the work to be done in training programs as well as program evaluation and the dissemination of findings. Funding was provided to help establish new change leadership roles (called CBME Leads) at each medical school [117].

### Creation of formal expert networks

While CBME had been discussed since the World Health Organization’s 1978 call to action [13], it was recognized when CBD started that many aspects of CBME implementation were still in development. Pooling of ideas and sharing of best practices and pitfalls would be a key ingredient in the project’s success. Therefore, the Royal College team founded and facilitated several national and international networks to facilitate knowledge creation and dissemination. These included the International CBME Collaborators, the Learning Analytics Medical Education Network, the Canadian Competence Committee Chairs Collaborative, a Residents Roundtable, a series of Program Evaluation Summits, Collaborations with the College of Family Physicians of Canada, and the medical school-based CBME Leads Roundtable.

### A priori program evaluation

As many aspects of CBD were new and transformative, it was an early priority to build a robust program evaluation strategy and network to ensure continuous improvement of all aspects of CBD. It was imperative that any negative unintended consequences of the new PGME system be recognized and ameliorated in a timely manner. Similarly, positive unintended consequences needed to be recognized, celebrated, and amplified. The CBD program evaluation strategy is elaborated in the paper by Hall et al. in this collection [118].

## The Competence by Design model: a bundle of 14 innovations to support a CBME system

Competence by Design involved transformational changes to all aspects of the Canadian specialist PGME system. Innovations were derived from a program logic model connecting the PGME issues and opportunities to the Van Melle Core Components of CBME [25] (see Table 2). All aspects of PGME, from core competencies to the role of time in training, to policies and standards for assessment, accreditation, credentialing, and certification, were reimagined from first principles. CBD “bundled “ 14 major innovations to enable the new PGME system, which are described below.

**Table 2:** Competence By Design Logic Model.


CORE COMPONENTS OF CBME*	ISSUES & OPPORTUNITIES	CBD DESIGN ELEMENTS	OUTPUTS	IMPACT

Outcomes as a Competency Framework for Graduates	PGME to ensure all graduates meet needed level of competence (focus on graduate outcomes for safe patient care) Program reviews focused on process, not outcomes	CanMEDS 2015 Framework New specialty-specific competencies New outcomes-oriented accreditation	Clear new competencies for every specialty New accreditation standards focused on outcomes of PGME	Competent graduates, ready for practice Enhanced training programs

Defined progression of training from novice to expert	Issues with transitions Time-based training produces variable graduates Patient safety concerns Incidents of inadequate supervision	Planned transitions 4 stages of PGME CanMEDS milestones	Better transitions to residency and practice Clear pathways to competence Better assessments for learning	Residents prepared for each stage of training Competent graduates, ready for practice Safer care

Tailored learning experiences	Generic training produces variable graduates Resident engagement with training enhances learning	Time variable training Flexible training requirements Promotions on achievements Individualized rotation plans Coaching over time	Residents with individualized pathways to certification	Residents prepared for each stage of training Competent graduates, ready for practice Greater resident satisfaction with training

Competency-based teaching	Little direct observation of trainees Inadequate feedback in workplace Growth mindset may enhance mastery of expertise EPAs provide opportunity for more faculty to provide better input Developmental view ensures no trainee left behind	Direct observation EPAs for workplace based assessment Coaching in the moment Developmental view of training Growth mindset	More direct observation More and better feedback Trainee portfolios provide rich picture of progress	Residents prepared for each stage of training Competent graduates, ready for practice Greater resident satisfaction with training

Programmatic assessment	Exam failures Promotions despite dyscompetence Few assessments Concerns about WBA Concerns about promotion decisions Opportunity to use learning analytics Opportunity to digitize assessment	Competence committee review of every trainee progress High number of EPA observations Learning analytics & eportfolios Developmental view of training Growth mindset Coaching over time New role for certification exam	Better promotion decisions Trainee portfolios provide rich picture of progress More faculty involved in WBA Clear pathways to competence Residents with individualized pathways to certification More and better feedback	Residents prepared for each stage of training Competent graduates, ready for practice Greater resident satisfaction with training Fewer appeals of assessments needed Same or higher exam pass rates


*After Van Mell E, et al. International Competency-based Medical Education Collaborators. A core components framework for evaluating implementation of competency-based medical education programs. Acad Med. 2019; 94: 1002–9.

### New competence framework with developmental milestones

The Royal College PGME system has used and regularly updated the CanMEDS competency framework as the basis of curriculum since 1996 [110, 111]. For CBD, a new version, CanMEDS 2015, was created that included developmental milestones for each domain of competence (e.g., communication skills) in the form of short statements that reflect a progression from the end of medical school to specialist level [88]. The milestones were deployed as a scaffold for workplace-based coaching conversations [119].

### Introduction of developmental entrustable professional activities

As described by Karpinski and Frank [120], the Royal College chose entrustable professional activities (EPAs) both as an approach to organize learning and as a framework for assessment. The CBD form of EPAs (RCEPAs) represented a series of professional tasks tailored to the specialty and the stage of training. They were explicitly developmental, in that RCEPAs grew in complexity and scope as training progressed. RCEPAs at the beginning of training were simpler (e.g., “Admitting patients to the Urology service”) and at the end of training reflected abilities approaching that of a practising clinician (e.g., “Coordinating, organizing, and executing the day’s list of surgical procedures”). An RCEPA included a description of the task, eight to 12 milestones from two or more CanMEDS Roles that are fundamental to complete the task, a supervision ordinal score (i.e., the O-Score [121]), and an area to complete a mandatory narrative comment. Such EPAs were to be directly observed in the workplace on a frequent basis, serving as a framework for monitoring progress (assessment of learning) and for coaching in the moment (assessment for learning) in the clinical setting [122]. RCEPAs were completed, logged, and aggregated in a digital platform. EPAs therefore served to: define progression of training, tailor learning of individual trainees, facilitate workplace based teaching around key tasks, and generate data for programmatic assessment.

### New stages of training

**Figure 2:**
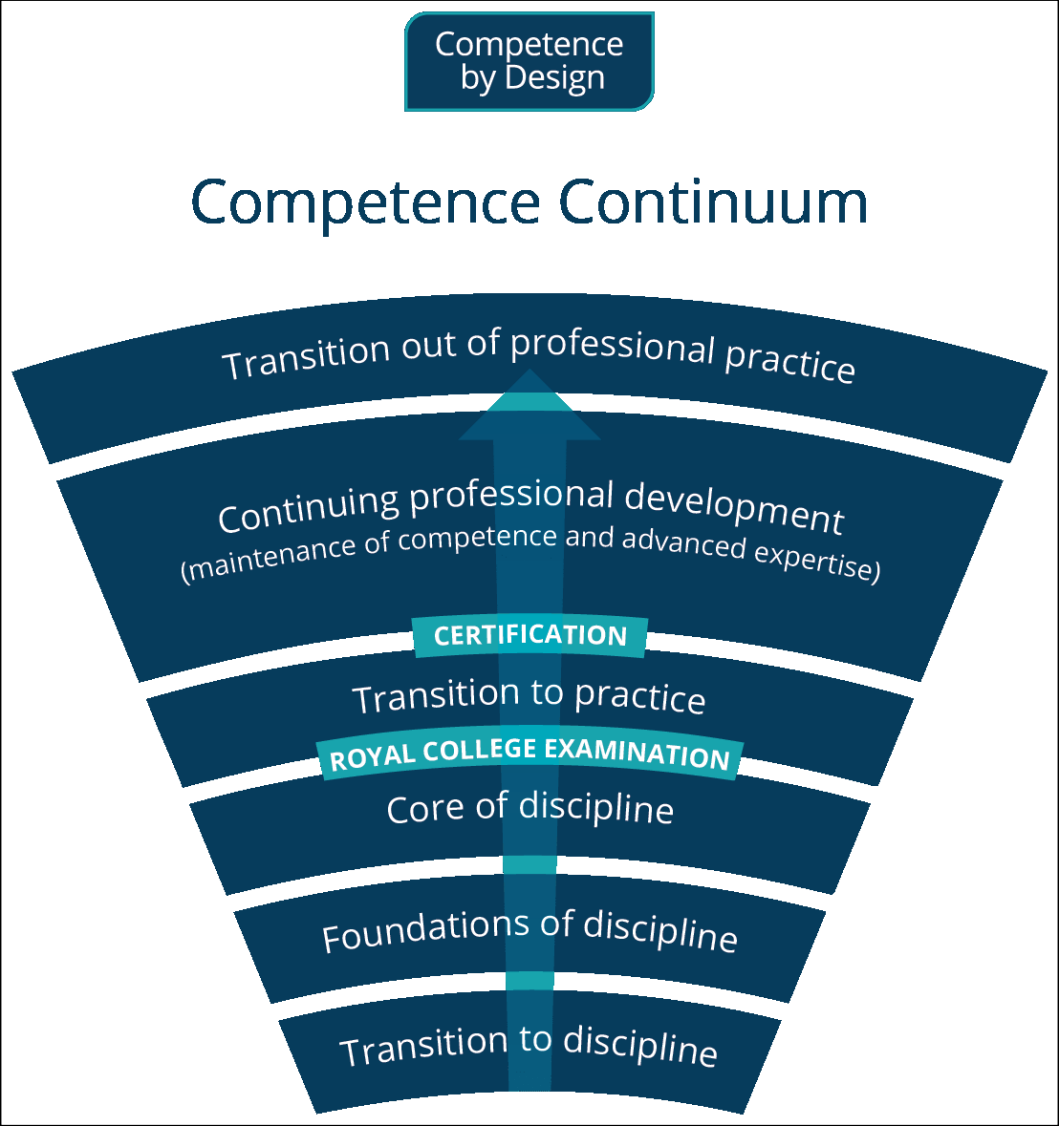
The Competence by Design Competence Continuum. Copyright 2012. The Royal College of Physicians and Surgeons of Canada. Reproduced with permission.

To enable a focus on program outcomes that ensures every graduate has acquired all of the competencies to practise safely, CBD moved from an organizing framework of time spent in training to competencies achieved sequentially [25, 103]. Postgraduate years (PGYs) were formally replaced in the educational system in favour of four defined stages of training: Transition to Discipline, Foundations, Core, and Transition to Practice. Each stage was designed to build upon previous experiences and achievements. Stages incorporated predefined competencies to be achieved, learning experiences (e.g., rotations, types of patient encounters, simulation sessions), EPAs, other assessments, and criteria for promotion. For the first time, specific attention was drawn to preparing trainees for transitions into PGME and into practice. Progression through the stages required a formal recommendation by the Competence Committee. The new standards required programs to prepare trainees for transitions between stages, ensuring they had acquired all relevant competencies, to increase their effectiveness on future rotations and promote safe patient care. The stages are illustrated in the CBD Competence Continuum (see Figure 2).

### New specialty-specific standards

The CanMEDS 2015 framework was used to template a new, updated set of competencies tailored to every Royal College specialty and subspecialty. Every discipline created a new national blueprint for curriculum and for assessment using these competencies, stages, and RCEPAs [123]. The disciplines then disseminated their new custom-built design for PGME based on the four stages of training; each stage included the requirements for training experiences, instruction, competencies to be achieved, and EPAs to be observed and recorded. These stages were not based on time or quotas for EPAs; time was a resource for learning, and workplace assessments were an opportunity to give/receive feedback and to document evidence of progress. CBD therefore provided a generational opportunity to revamp each specialty’s training design.

### New program blueprints for teaching and assessment

The CBD national specialty standards were translated into new blueprints for teaching and for assessment at all of the nearly 1000 Royal College training programs in Canada. These were required as an accreditation standard. Local program committees were asked to use this opportunity to reimagine training in their context, allocating time to essential rotations and instruction and allocating EPAs and other assessments to planned experiences. This provided an occasion to reassess the best experiences to aid achievement of competence and reconsider experiences that were no longer needed. In this way CBD facilitated renewal of every training program in the country.

### Workplace-based assessment system with direct observation

Competence by Design introduced a new workplace-based assessment system that placed emphasis on both assessment *for* learning and assessment *of* learning [68, 69]. Instead of a single retrospective workplace-based assessment completed at the end of every four weeks of training by a single supervisor, programs were asked to ensure that every trainee was frequently observed using EPAs in the workplace and received coaching in the moment around that EPA, and that the supervisor’s impressions were recorded in an electronic portfolio. EPAs were employed to ensure multiple micro-assessments of performance, captured from multiple assessors to inform a richer and more reliable determination of learner progress. In a study of the first year of implementation in a single specialty, observations recorded per trainee rose from fewer than 20 traditional assessments to 90–230 EPAs across sites [124].

### Competency-based coaching model

To address concerns around a lack of useful feedback given to trainees in traditional training [125], EPAs in CBD were not solely used as an assessment framework. EPAs were also the foundation of frequent direct observations [126] and clinical coaching in the moment [127, 128]. To support this, the “RX-OCR” coaching model was rolled out, as described by Richardson et al. in this collection [122].

### Training in a growth mindset

The introduction of increased direct observation, workplace-based assessment, and competency-oriented coaching feedback created the risk that the new CBD system would be overwhelmingly focused on assessment. Instead, the goal of the new system was to enhance teaching and learning [101]. Therefore, the CBD organizers explicitly included orientation for trainees and teachers to Carol Dweck’s growth mindset [129, 130]. This approach to competency-based education advocates a developmental view of learning; every learner is on a journey to competence, and any given competency is achieved or *not yet achieved*. Under CBD, teachers were encouraged to record not just when a trainee achieved an EPA or area of competence but also their progress enroute. Many observations meant that any given data point in a learner’s portfolio was a “pixel in a picture of competence,” and each one was not high stakes. The goal was for trainees and teachers to use discussions of trainee abilities as a “progress note” on development and not a commentary on a learner’s character. This was a distinct shift from the fixed mindset that is prevalent in medical education [100, 122, 128].

### Introduction of competence committees and programmatic assessment

Programmatic assessment was incorporated to synthesize a spectrum of assessments (mainly RCEPAs but addressing various assignments relevant to the stage of training) from a diversity of supervisors over time into a global assessment of a trainee at a specific stage of training. In the CBD model, a Competence Committee was a formally appointed group of dedicated educators who met regularly to look at the EPA and other aggregated performance data in a trainee’s portfolio and assign a formal status to their progress (e.g., “Progressing as Expected”) using a prescribed consensus process. Feedback and an educational prescription were to be provided to the trainee. Modification of future training was also possible, including early remediation or accelerated progression through training. The Competence Committee reported to the overall Residency Program Committee (RPC), and the RPC was accountable for and aware of all promotion recommendations and decisions to ensure overall alignment across the local program [131,132,133,134].

### Introduction of electronic portfolios

CBD made digital trainee portfolios an essential ingredient in residency education [135]. The Royal College wanted to move away from the use of paper forms (still present in some programs) and incentivize the use of more sophisticated electronic portfolios to manage the increase in trainee progress data (e.g., EPA observations) [78,79,80]. Digital completion of RCEPAs ensured efficient and secure data capture. An electronic portfolio allowed for dashboard views that trainees, teachers, and Competence Committees could use to monitor trainees’ progress in meeting the program requirements [136, 137]. To support all of this, the Royal College invested in and provided a free eportfolio for every accredited program. Universities and hospitals also had the option of implementing another electronic portfolio of their choice.

### Learning analytics

CBD enabled learning analytics nationally for the PGME system with the availability of many more assessment data points and ease of aggregation of digital data. While learning analytics are prevalent in higher education [138], there was minimal use in Canadian PGME before the CBD rollout. Learning analytics are a powerful set of tools to display trainee progress against a standard. These analytics may also provide views on teacher behaviour, rotation effectiveness, and the program overall [139]. A powerful graphical learning analytics dashboard has become an important tool for Competence Committees under CBD [81, 108].

### Changed role for certification examination

For many decades, the Royal College certification examination was the final act of PGME, occurring at the end of training as a single, high-stakes external gateway to independent licensure and certification. Under CBD, this examination was moved to the end of the Core stage of training [140]. This action moved examination preparation, a powerful driver of learning, into an earlier stage of training and created an examination-free period of time for a true Transition to Practice stage. In general, examination candidates performed just as well when examinations were moved from the end of training to the end of the Core stage. The examination became another major exhibit in a trainee’s portfolio of progression to competence, and successful completion of the examination was still a requirement for certification. The role of examinations in CBD is further elaborated in Bhanji et al. in this collection [141].

### New accreditation standards emphasizing continuous quality improvement and quality assurance

To support the rollout of the CBD system, the Royal College accreditation system was also renewed to focus more on outcomes. As part of a consortium of accreditation stakeholders (CanRAC), a fresh set of standards was produced that included requirements around the elements of CBME [142]. In keeping with the philosophies that inform CBD, the new accreditation system shifted its emphasis from quality assurance (meeting a standard) to continuous quality improvement (rewarding programs showing a strategy of enhancing the program) [106]. The new accreditation system for CBD is further described in the accompanying paper by Dalseg et al in this collection [143].

### Time-variable credentialing

Finally, CBD marked a move away from credentialing for certification based on time spent in training on prescribed clinical services [144]. Instead, the Royal College adopted a policy of accepting the promotion decision of a local program Competence Committee based on all of the data available on a trainee’s readiness for practice [132]. As a safeguard, Competence Committee functions were examined as part of accreditation visits. While CBD represents a hybrid time-variable approach, trainees in CBD programs could graduate earlier than the standard training duration if there was evidence that they had achieved all required competencies and training experiences.

## Discussion

While many countries have begun work on CBME designs, little has been published to date describing a national-scale transformation of a PGME system to competency-based education. In this paper, we have described CBD as a unique innovation in health professions education, and elaborated the drivers, development, and design of a novel CBME system that was the biggest change in Canadian training since the founding of PGME in the country. While it may be that all educational programs are continuously evolving, large-scale transformations in education systems are not common [145]. The CBD project represents both a transformational change to an existing PGME system and an application of time-variable CBME. There are several lessons learned from the early implementation of CBD and implications for those leading change in health professions education.

### Lessons learned about large-scale educational change

#### Large-scale change provides a rare opportunity to reimagine how a system works

The CBD initiative became an opportunity to fundamentally reimagine every aspect of a PGME system, from policies to philosophies to procedures, from accreditation to assessment. This is a rare phenomenon in professional education. This allowed many long-standing concerns and ideas to be addressed as part of this implementation (e.g., digitizing accreditation reviews). Those involved were committed to continuous improvement, ultimately for future graduates and their patients. Nevertheless, the occasion to re-examine fundamentals must be balanced with the high degree of effort needed to pursue such an opportunity.

#### Organizational transformation is needed to sustain “big change”

In the course of implementing CBD, it was realized that to be successful, the participating organizations themselves needed to change. Organizations changed policies, procedures, and personnel. The latter change was necessary to put in place individuals dedicated to new educational processes. Fundamentally, CBD changed the mental models of all those who adopted this new way of preparing physicians, including the leadership of the institutions involved [146].

#### Co-production with stakeholders is essential

The implementation of CBD required the engagement of numerous stakeholders to make progress in change. Stakeholders such as trainee organizations and faculties of medicine were on the front line of impacts of any PGME project, so they had critical input into shaping CBD. Co-production of the elements of CBD with partners, while slower, was essential to get the best possible design from many perspectives [115].

#### An adaptive program evaluation strategy is essential

From the outset, CBD developed a robust program evaluation strategy with three main pillars of activity: readiness to implement, fidelity of implementation, and outcomes. Ongoing evaluation studies from across the PGME system rapidly informed education leaders of issues, concerns, strengths, and regional variations. This was absolutely critical to the success of such a large and complex change project [118].

#### Large-scale change in medical education can lead to scholarship and career changes

Anecdotally, CBD triggered participants to change roles and produce scholarship, as an unintended impact of the transformation. Trainees and faculty became interested in an education career track, becoming chief residents (chief postgraduate trainees), program directors, or scholars.

### Lessons learned about CBME implementation

#### CBD is a CBME proof of concept at scale

CBD was explicitly created to use the Van Melle Core Components of CBME [25]. Among the criticisms of the CBME movement, there has been a concern that this approach is theoretical, without an evidence base or track record [84]. CBD contributes to the discourse of HPE by demonstrating that the Core Components of CBME can be used as the basis for a 21 st century national PGME system.

#### There are benefits to implementing CBME as a “bundle” of changes

In previous work by the International CBME Collaborators, many pioneering CBME designs focused primarily on competency frameworks or programmatic assessment. These are two of the Van Melle Core Components. By contrast, the CBD initiative used all of the Core Components, which led to 14 implemented innovations “bundled” into one transformative system change [147, 148]. Early program evaluation findings suggested that greater alignment with the changes at the training program level produced better alignment with the desired outcomes of the CBD Logic Model [118].

#### Time is a resource for learning, not the criterion for completion of training

PGME systems use time in training in a variety of ways. Some have fixed-time designs that require a specified number of weeks in prescribed learning experiences. Systems based on time spent have been criticized for having the potential risk that graduates may exit without having achieved all required competencies for safe practice. By contrast, open-time systems have been criticized for inefficiency and prolonged training. CBD tried to create a time-hybrid system, with guidelines for learning experiences that enable achievement of required competencies. In this system, time is a resource for training, not the organizing framework. Therefore, given rotations were recommended, not required, and the achievement of competence was not based on time. Quality controls were built into accreditation visits to ensure programs were tailoring training to individual trainees’ needs, while ensuring there was evidence that every graduate had achieved all essential competencies [144].

#### Time variability enabled individualized learning plans

The CBD design allowed individualized trainee learning plans. Competence Committees were encouraged to consider future training experiences on the basis of what the trainees’ portfolios indicated they needed to progress to the next stage. For the vast majority of trainees, this did not mean early or late completion of training. What it did mean was that Competence Committees were able to recommend, as needed, changes to a trainee’s rotations or other activities to enable them to achieve the program outcomes. In doing so, programs balanced the needs of clinical services and the trainee’s educational needs. The extent to which this was implemented varied [134].

#### “Developmental” EPAs facilitate progress decisions

Many health professions education programs around the world that use EPAs have designed them to be tasks that a graduating trainee works toward. In CBD, the Royal College explicitly wanted to sequence training from novice to expert and ensure learners truly were prepared for each stage of their development. This aligned with the theory of the Core Components, addressed concerns about patient safety by ensuring trainees were prepared for their tasks, and allowed for direct observation and coaching around specific tasks for the level of training. By pinpointing tasks that a learner was expected to be able to perform at the end of each stage, Competence Committees had a set of criteria to guide promotion decisions [120].

#### Better feedback is possible

One of the drivers for CBD was perennial complaints about the lack of useful and actionable feedback to trainees [62,63,64,65,66,67]. The strategies chosen to address this included the deployment of EPAs as a focus for learning and observation, a new workplace coaching model, explicit discussion of the growth mindset, and requirements for regular direct observation and coaching feedback. Early evaluation studies showed that trainees reported more frequent and more actionable feedback as part of workplace-based assessments and EPA conversations [118].

#### Programmatic assessment offers key benefits

A fundamental pillar of CBD was the deployment of programmatic assessment. Its use was intended to address long-standing concerns about PGME assessment being subjective, lacking a comprehensive view of development of competence, and being based on too few supervisors and/or too few observations. Programmatic assessment was a major change for most programs in the PGME system, with variable rates of adoption. In the programs where this approach to assessment was adopted with fidelity, local education leaders reported high satisfaction with stronger assessment decision-making, richer data on individual trainees, fewer appeals of assessment decisions, and better quality feedback to learners. When programmatic assessment was conducted, summative assessment decisions were shifted from the workplace supervisors to Competence Committees [133, 134, 149, 150].

#### Real-time, low-stakes workplace-based assessment is possible

Worldwide, a major challenge to supporting a robust CBME design lies in obtaining an adequate number of useful direct observations in the workplace of trainee progress toward competence [45, 126]. In implementing CBD, the Royal College asked supervisors at all 1000 training sites to sample every trainee’s work on a regular basis. It was found that supervisors can do direct observation in small, brief episodes and record rich and useful notes on trainees. This was not easy for all settings, but some programs did realize a shift towards greater direct observation [118, 150].

#### Competence Committees work

In CBD, program leaders reported high satisfaction with implementing Competence Committees [118, 133]. They reported that individual trainees were discussed in greater depth and richness, that assessment decisions were more robust, and that the processes to create a functional formal Competence Committee were doable across multiple settings. This experience provides further support for the use of Competence Committees in PGME.

#### CBME may support equity in assessment

Recently, multiple studies have identified concerns with equity in assessment of trainees in various Refs. By requiring that all trainees — not just those favoured or flagged for concern — be directly observed by multiple supervisors and discussed at a Competence Committee on a frequent basis, the CBD assessment system took a small step toward equity.

#### Pitfalls in PGME transitions can be ameliorated

Multiple previous reports flagged that transitions in training are stressful for learners, could put patients at risk, and are not ideal education designs [39,40,41,42,43, 102]. These transitions — from medical student to PGME trainee, from junior to senior trainee, and from senior trainee to practice — are perennial challenges. CBD explicitly planned to address these challenges by using stages as a deliberate sequence of training. In particular, the Transition to Discipline stage explicitly oriented the learner to the discipline and promoted the learner’s professional identity development as a junior member in that discipline. The Transition to Practice stage provided a capstone opportunity for the trainee to safely act in the role of the most responsible physician or surgeon while preparing for the realities of independent practice.

#### Learning analytics is a powerful suite of tools with benefits

While learning analytics has existed in education for a long time, adopting programmatic assessment under CBD allowed the first whole-scale use of this suite of tools across a PGME system. Learning analytics allowed trainees to visualize their progress, Competence Committees to make data-driven decisions, faculty to improve their feedback, programs to gain insight on learning environments, and institutions to flag outlier programs [80, 81, 136,137,138,139, 151,152,153,154].

#### Certification examinations still have a role

Under CBD, the Royal College certification examination was maintained and moved to become a formal assessment after the Core stage of training. Pass rates were, on the whole, unchanged. From an educational design perspective, the earlier certification examination was considered by many to be a powerful driver for learning, to be another key data point for Competence Committees, and to enable a focus on transition to practice after the examination was completed [141].

### Lessons learned: pitfalls in large-scale CBME implementation

The implementation of CBD is ongoing. At every step of the change, challenges were encountered that have potentially important implications for others contemplating CBME and other transformational education changes.

#### Large-scale change stresses a system

CBD brought 14 innovations to a national PGME system. Inevitably, some training sites found the changes easier to adopt than others. On the basis of the accreditation achievements across training sites, the CBD design team assumed the new design was achievable by all 1000 training programs. However, once change was underway, some training sites reported difficulties with various aspects of the new training scheme. Some of these reported difficulties had been existing requirements of the previous training paradigm; the transformation to CBD shed new light on challenges within the PGME system. Multiple institutions reported greater cost to implement CBD than expected. Variability across the country was the biggest pattern, and some presumed training features were not always in place when CBD arrived.

#### Specialty variability required flexible approaches

Medical disciplines (specialties, subspecialties, etc.) have their own distinct subcultures. As CBD rolled out, disciplines displayed differing levels of responsiveness to change, ability to undertake educational reform, and cohesiveness. The CBD team worked with disciplines individually to support the rollout of the new training approach. Clinical realities (e.g., the COVID pandemic, resource stressors) seemed to impact education adoption [116].

#### Requirements for workplace-based assessments were a wellness issue

One unexpected development early in CBD was trainee stress with the new programmatic assessment requirements. Guidelines related to EPA observations to populate each learner’s portfolio were perceived as quotas, and residents were often given the responsibility to initiate faculty engagement in EPA form completion. These implementation issues led to some training sites reporting wellness issues with trainees that were not anticipated. While we hoped teaching Dweck’s growth mindset and a learner-centered approach would help trainees see the new workplace-based assessments as beneficial, this was clearly not universal in the early years of the new scheme [53, 98, 150, 155].

#### Implementing large-scale change during a pandemic was unanticipated

The implementation of CBD was planned as a multiyear project, and the COVID-19 pandemic began when CBD implementation was underway. As with HPE worldwide, CBD designs were drastically impacted [156]. Not only were certain learning experiences shut down for periods, but trainees and teachers were redeployed to treat large numbers of patients with COVID-19. Fortunately, the flexibility built into CBD allowed trainees to continue to progress in their training, employing evidence of achievement of competencies from alternative activities.

#### Electronic portfolio technology was problematic

At the time CBD was conceived, it was assumed that a country with a small population like Canada would share a national eportfolio developed by the Royal College and deployed for free. However, it was soon found that no software package satisfied all the needs of training centres, met preferred workflows, or was deployable in every software environment. In addition, trainees and institutions raised learner privacy concerns, so there were unexpected barriers with data sharing [157]. Instead, numerous local electronic portfolios were used across the country over time and the landscape continues to evolve rapidly.

#### Growth mindset is difficult to implement across PGME

As discussed above, one of the innovations of CBD was to encourage adoption of Carol Dweck’s growth mindset approach to teaching, learning, and assessment across the PGME system. Early in CBD, participants were intrigued, but widespread adoption was not readily seen. Instead, embracing a new mindset was an innovation that appeared to be on a long, slow adoption curve [101].

#### Competencies can be subsumed when using EPAs

CBD promoted the use of both CanMEDS competencies and EPAs as dual frameworks. However, in promoting EPAs as part of supporting implementation, educators on advisory committees reported a concern about over-emphasis on EPAs. As an unintended consequence, there was a perception of less emphasis on CanMEDS in PGME than before CBD.

## Comparisons to Other CBME Implementations in PGME

In a 2021 study by the International CBME Collaborators, the majority of CBME programs surveyed had worked on implementing two of the van Melle Core Components: a competence framework and programmatic assessment [158]. However, four major PGME programs were comparable in scope and scale to Competence by Design: the Triple-C project of the College of Family Physicians of Canada [27, 159, 160], the ACGME Outcomes project in the US [22], the Australian Orthopaedic Association’s AOA-21 curriculum [161], and the Dutch Association of Medical Specialties’ Individualizing Postgraduate Medical Training project [162]. All of these competency-based PGME initiatives feature their own methods for implementing the van Melle Core Components, as in CBD. A simple comparison of the design features of these initiatives is displayed in Table 3.

**Table 3:** Comparing CBD to Other CBME Implementations.


CORE COMPONENT	COMPETENCE BY DESIGN(ROYAL COLLEGE OF PHYSICIANS AND SURGEONS OF CANADA)	TRIPLE-C(COLLEGE OF FAMILY PHYSICIANS OF CANADA)	OUTCOMES PROJECT(ACCREDITATION COUNCIL FOR GRADUATE MEDICAL EDUCATION, USA)	AOA 21(AUSTRALIAN ORTHOPAEDIC ASSOCIATION)	INDIVIDUALIZING POSTGRADUATE MEDICAL TRAINING(DUTCH ASSOCIATION OF MEDICAL SPECIALISTS, NETHERLANDS)

Training outcomes organized as a competency framework for graduates	CanMEDS framework	CanMEDS-FM framework	ACGME 6 Competencies	AOA 21 Curriculum Framework	CanMEDS framework

Defined progression of training from novice to expert	Stages of training	Progression through training program	ACGME Milestones	Stages of training	Postgraduate years and EPAs

Tailored learning experiences to meet the needs of learners	Time-variable, flexible training	Tailoring within program	Tailoring within program	Time-variable, flexible training	Time-variable, flexible training

Teaching focused on competency achievement	EPA-driven, direct observation, and coaching in workplace. Growth mindset.	Teaching guided by Assessment Objectives for Certification in Family Medicine	Teaching guided by ACGME milestones	Teaching focused on stage-specific curriculum	Teaching focused on EPAs

Programmatic assessment	CBD program of assessment including Competence Committee review. Multiple eportfolios.	Triple-C program of assessment including Continuous Reflective Assessment for Training (CRAFT) reviewed by residency program committee. Multiple eportfolios.	Milestones-based program of assessment including Clinical Competency Committee review Multiple eportfolios.	AOA-21 program of assessment including Regional Training Committee review. National eportfolio.	EPA-based Program of assessment including Clinical Competency Committee review. Multiple eportfolios.


All of these initiatives reported similar challenges implementing large-scale change into a PGME system. These initiatives each required major change management efforts and resources. Every one of these transformative curriculum changes required major investments in faculty development immediately for implementation [163] (e.g. the Dutch curriculum alone reached ~7000 clinical supervisors [164]). All of them reported stakeholders’ concerns with the new workplace-based assessments [52, 53, 150, 155, 165, 166], though only the Dutch system and CBD required the use of EPAs. They also shared initial challenges with digital assessment portfolio software that improved over time. All groups revised their assessment requirements based on feedback from concerned stakeholders.

There were some benefits in common as well. All of the reported enhanced feedback opportunities for trainees. All of these initiatives successfully deployed competence committee-type programmatic assessment of trainees which increased the rigour of judgments about competence [77, 83, 133, 134, 149]. Time-variable, trainee-tailored training was achieved to varying degrees in the Netherlands, AOA-21, and in CBD, while finding ways to ensure service provision was not overly impacted. (Time-variability was not a design element of Triple-C and ACGME.) Overall, all of these major competency-based PGME curricula for the 21st century were successfully deployed, sustained, and evolved over time [93, 159,160,161,162, 167,168,169].

## Limitations

As discussed above, this paper is written from the perspective of the Royal College design and implementation team in place at the time. It describes the data available to this team. With any such large-scale transformation, there are inevitably differing perspectives from a variety of stakeholders. These often vary over time, vary with the issues in question, and vary with the degree of intensity of emotion involved. CBD was no exception. As CBD evolved, other PGME stakeholders and commentators had differing perspectives. Each of these perspectives has lessons for change leaders in HPE.

## Conclusions

Competence by Design is a major transformational change to a national postgraduate medical education system. A bundle of 14 innovations, CBD provides an example of implementation of competency-based time-variable outcomes-oriented medical education at scale. CBD addresses recurring concerns about 20^th^ century training designs that can impact patient care provided by graduates. Others interested in implementing CBME can learn lessons from the CBD design and experience.

## Additional File

The additional file for this article can be found as follows:

10.5334/pme.1096.s1 Supplement A. Phases and activities of the Competence by Design project.
