# Supplementary material for: Competence By Design: a transformational national model of time-variable competency-based postgraduate medical education
Source: Perspect Med Educ. 2024 Mar 18;13(1):201–23. doi: 10.5334/pme.1096 (PMC10959143; doi:10.5334/pme.1096)
Supplement: Supplement A. — Phases and activities of the Competence by Design project. [file pme-13-1-1096-s1.pdf]

Supplement A: Phases and activities of the Competence by Design project

**Major activity**	**Phase 0**<br>**Plan the plan**<br>**(2010–2012)**	**Phase 1**<br>**Preparation**<br>**(2012–2015)**	**Phase 2**<br>**Implementation launch**<br>**(2015–ongoing)**	**Phase 3**<br>**Evaluation and adaptation**<br>**(2010–ongoing)**
**Organizational alignment and support**	• Project team<br>• Project charter	• Formal project plan<br>• Resource allocations<br>• Working groups<br>• Networks	• Implementation oversight<br>• Coordination<br>• Change support	• Evaluation oversight<br>• Coordination<br>• Change management
**Design: iterative community development and rollout**	• Logic model<br>• CBME model	• CanMEDS 2015<br>• EPA pilot projects<br>• Accreditation renewal	• Rollout by specialty cohort	• Ongoing rollout<br>• Revisions
**Policy**	• Impact evaluation	• Policy revision and consultation	• Policy approvals	• Policy monitoring and revisions
**Standards**	• Impact evaluation	• Specialty standards renewal development	• Standards workshops<br>• Standards rollout	• Standards workshops<br>• Revisions
**Partnerships:** stakeholder engagement and coproduction	• Engagement	• Co-production<br>• Consultations	• Co-production<br>• Consultations<br>• Shared leadership<br>• Shared resources<br>• Expanded engagement	• Harvesting concerns<br>• Co-production<br>• Consultations<br>• Shared leadership<br>• Shared resources<br>• Expanded engagement
**Evaluation**	• Evaluation principles and design	• Three pillars: readiness, fidelity, and outcomes<br>• Study designs<br>• Stakeholder consultation<br>• Evaluation team	• Data collection, analysis, reporting<br>• Sharing of lessons learned<br>• Stakeholder collaboration	• Responsive adaptation<br>• Challenge clarification<br>• Knowledge translation

CBME competency based medical education; *EPA* entrustable professional activity
